# COVID-19 and mental health: a longitudinal population study from Norway

**DOI:** 10.1007/s10654-021-00836-3

**Published:** 2022-01-27

**Authors:** Hans K. Hvide, Julian Johnsen

**Affiliations:** 1 University of Bergen, Bergen, Norway; 2 CEPR, London, UK; 3 University of Aberdeen, Aberdeen, UK; 4 SNF—Centre for Applied Research at NHH, Bergen, Norway; 5 FAIR, Bergen, Norway

**Keywords:** Primary care, Mental health, Psychological disorder, Psychological symptoms

## Abstract

Existing research has found adverse short-term effects of the COVID-19 pandemic on mental health, but longer-term effects have been less documented. Using newly released register data on all general practitioner consultations in Norway through 2020 (about 14 million consultations in total), we find that during the spring and early summer 2020, the number of psychological cases initially increased relative to prior years, but then fell back towards the level of prior years during the summer 2020. In early September 2020, the number of cases accelerated, a pattern that held up through December 2020, so that the gap between 2020 and prior years became largest end-of-year. Our findings suggest that the accumulated adverse effects of the COVID-19 pandemic on mental health far exceeds the short-term effects. The effects are particularly strong for females and for residents in urban areas.

**Supplementary Information:**

The online version contains supplementary material available at 10.1007/s10654-021-00836-3.

## Introduction

Many researchers have investigated the short-term consequences of the COVID-19 pandemic on mental health problems. Survey evidence from several countries suggest that the fear of infection and death from COVID-19, income insecurities, and limit to personal freedoms led to an increase in depression, anxiety, and substance abuse in the spring and early summer of 2020 [1–4]. Evidence on the longer-term effects is scarce [5]. People may have developed better coping strategies, but the accumulated effects of stress may take its toll. We used newly released register data covering the universe of general practitioner (“GP”) consultations in Norway until the end of 2020 to address the longer-term consequences of COVID-19.

## Materials and methods

Data from the Norwegian Control and Payment of Health Reimbursement register (KUHR) form the basis of the analysis [6]. The KUHR data we used cover all patient encounters with GPs in Norway in the years 2017–2020. Each row in the KUHR data consist of a single encounter and includes one or more codes classifying the patient’s condition. In addition, the KUHR data contain the date, time, and type of encounter.

The diagnostical codes in KUHR are according to the ICPC-2 classification system (International Classification of Primary Care) developed by WONCA (World Organization of Family Doctors) in 1987. ICPC-2 is a classification method for primary care encounters that includes codes both for the patient’s reason for encounter and for diagnoses. The psychological codes P01-P99 are divided into symptoms and complaints (P01-P29) and diagnoses (P70-P99). For example, P03 “Feeling depressed” is a symptom/complaint and P76 “Depressive disorder” is a diagnosis.^1^ We refer to any P-code being assigned by the GP as a P-case. Furthermore, we refer to P01-P29 as “non-severe” cases and P70-P99 as “severe” cases.

The total number of GP consultations in 2020 was about 14 million, and about 1.8 million (14%) resulted in a P-case.^2^ The percentage of the population that consulted a GP in 2020 was 75%, identical to the prior years (see Table S1). Due to a fast transition to electronic consultations in Norway, the GP system did not experience a large drop in encounters in the months after the COVID-19 outbreak in March 2020 (see the Supplementary Appendix). This was quite different from e.g., the UK [7] and the US [8].

We merged the KUHR data with sociodemographic registers, also covering the whole population, using the unique person ID. The ID is an anonymized version of an individual’s social security number. This allowed us to merge in gender, age, and municipality of residence variables for each patient.

As outcome variable we used the number of weekly P-cases, calculated between January 1 and December 31, 2020. As comparison group, we used the number of weekly P-cases averaged over the years 2017–2019. We analyzed both percentage increases and increases per capita, population-wide and for subpopulations. We also analyzed the increase in cases separately for severe and non-severe P-cases, and for the eight most common psychological symptoms/diagnoses in 2019, i.e., pre-pandemic.

We performed two robustness checks: First, we controlled for a possible “2020 effect” unrelated to COVID-19 by comparing the increase in average weekly cases during weeks 40–51 in 2020 to the corresponding increase during weeks 1–10 of 2020 (i.e., prior to the outbreak). Second, to investigate whether the COVID-19 effects interacted with a potential “long winter” effect, we analyzed the increases in P-cases for the three northern-most counties (Nordland, Troms, and Finnmark).^3^ Here, the population live close to or above the arctic circle.

Poisson regressions were used to assess statistical significance. We regressed the average number of weekly cases in week 40–51 on a dummy for year 2020. The coefficients of the regressions can be interpreted as percentage increases from 2017–2019 to 2020.

All analysis was performed using Stata version 16.1. To define weeks, we used Stata’s inbuilt time functions. As week 52 in Stata has different length in different years, it was excluded from the analysis (the gap between 2020 and 2017–2019 is larger in week 52 than in prior weeks). By “population” (capita) in Fig. 2 we mean the individuals that attended their GP during the year, i.e., around 75% of the total population of Norway. The figures use 3-week moving averages for the outcome variables.

## Results

**Fig. 1:**
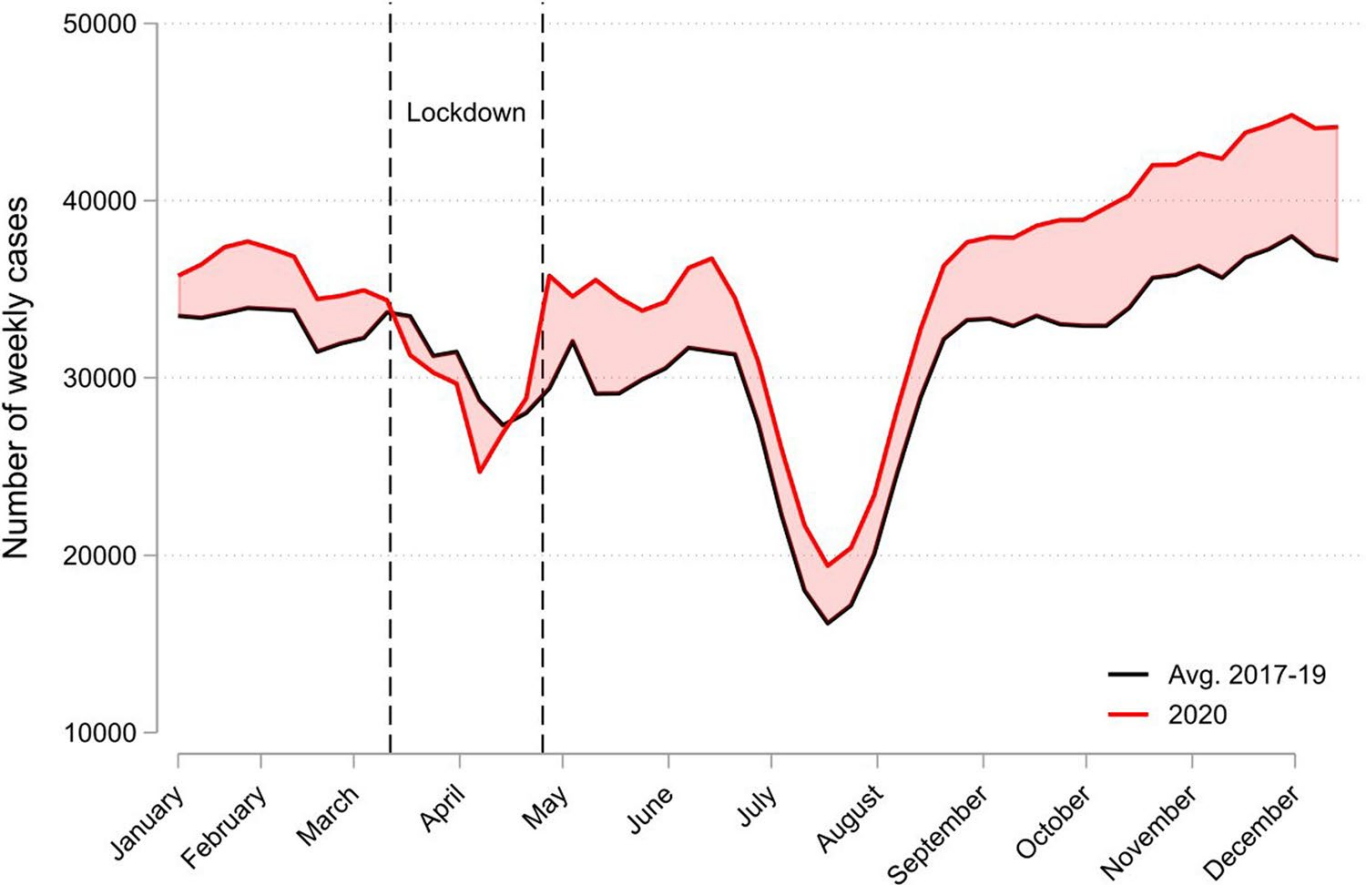
Weekly number of P-cases in 2020 (red line) versus 2017–2019 average (black line). *Note* A “P-case” is a GP consultation that related to a psychological symptom, complaint or diagnosis based on the ICPC-2 classification system (P00-P99). The Figure uses 3-week moving averages for the outcome variables. The leftmost vertical dashed line indicates March 12th, the start of both the first serious outbreak of coronavirus in Norway and the start of national infection control measures, while the rightmost vertical dashed line indicates the end of the strictest measures (e.g. closure of schools and psychologists) on April 27th. Other measures such as social distancing, remote teaching at universities, and remote work were in place throughout most of the period after March 12th

Figure 1 depicts the population-wide weekly P-cases for 2020 (red line), using the 2017–2019 average (black line) for comparison. After the COVID-19 outbreak in March 2020 (leftmost vertical dashed line), the number of P-cases in 2020 became larger than the 2017–2019 in late spring, but more similar during summer. In early September, the gap between 2020 and 2017–2019 started increasing, a pattern that held up through December 2020.

Table 1 reports the number of cases in September-December 2020 (weeks 40–51), compared to the same period in 2017–2019. Panel A shows that the increase in P-cases in 2020 was about 17% [95% CI 0.16–0.19] relative to 2017–2019. For non-severe P-cases, the increase in 2020 was about 22% (95% CI 0.20–0.24), while for severe P-cases the corresponding increase was about 13% (95% CI 0.11–0.15).

**Table 1:** P-cases in week 40–51, 2020, versus average P-cases week 40–51 in 2017–19

	Avg. number of weekly cases			Output from Poisson regression		
	2017–2019	2020	Difference	Coeff	95% CI	*p* value
*Panel A. All (age 11* +*)*						
P-cases	35,610	42,387	6777	0.17	0.16–0.19	< 0.001
Non-severe P-cases	16,276	20,359	4083	0.22	0.20–0.24	< 0.001
Severe P-cases	20,060	22,912	2852	0.13	0.11–0.15	< 0.001
P-cases, controlling for week 1–10				0.09	0.07–0.11	< 0.001
*Panel B. Subgroup P-cases*						
Age 11–17	1444	1807	363	0.22	0.16–0.29	< 0.001
Age 18–30	7253	8356	1103	0.14	0.11–0.17	< 0.001
Age 31–64	21,909	25,872	3963	0.17	0.15–0.18	< 0.001
Age 65 +	5004	6352	1348	0.24	0.20–0.28	< 0.001
Male	13,631	15,682	2051	0.14	0.12–0.16	< 0.001
Female	21,979	26,705	4726	0.19	0.18–0.21	< 0.001
Urban	5002	6219	1217	0.22	0.18–0.25	< 0.001
Rural	25,605	29,949	4344	0.16	0.14–0.17	< 0.001
Northern-most counties	2498	2737	239	0.09	0.04–0.15	0.001
*Panel C. 8 most common psychological diagnoses/symptoms*						
P01 Feeling anxious	2657	3194	537	0.18	0.13–0.24	< 0.001
P02 Acute stress reaction	3945	4724	779	0.18	0.14–0.22	< 0.001
P03 Feeling depressed	1570	2023	453	0.25	0.19–0.32	< 0.001
P06 Sleep disturbance	3380	4406	1026	0.27	0.22–0.31	< 0.001
P29 Psych. symptom other	3608	4321	713	0.18	0.14–0.22	< 0.001
P73 Affective psychosis	1225	1453	228	0.17	0.09–0.25	< 0.001
P74 Anxiety disorder	2963	3677	714	0.22	0.17–0.26	< 0.001
P76 Depressive disorder	8980	10,235	1255	0.13	0.10–0.16	< 0.001
P81 Hyperkinetic disorder	1420	2044	624	0.36	0.30–0.43	< 0.001
P82 PTSD	1263	1761	498	0.33	0.26–0.40	< 0.001

A “P-case” is a GP consultation that related to a psychological symptom, complaint or diagnosis based on the ICPC-2 classification system. In row 4, we used four observations: average weekly cases for week 1–10 in 2017–2019, average weekly cases for week 40–51 in 2017–2019, average weekly cases for week 1–10 in 2020, and average weekly cases for week 40–51 in 2020. Using this sample, we ran a Poisson regression, regressing average number of weekly cases on a dummy for year 2020, a dummy for week 40–51, and the interaction of year 2020 and week 40–51. We report the coefficient of this interaction, which can be interpreted as the extra percentage increase in average weekly cases from 2017–2019 to 2020 compared to the increase in average weekly cases from 2017–2019 to 2020 for the pre-Covid part of the calendar year

Panel B of Table 1 shows that the largest percentage increase was for age 11–17 (0.22; 95% CI 0.16–0.29), age 65 + (0.24; 95% CI 0.20–0.28), for females (0.19; 95% CI 0.18–0.21) and for urban (0.22; 95% CI 0.18–0.25), the latter being inhabitants of the four main cities (Oslo, Bergen, Trondheim, Stavanger).

Panel C of Table 1 shows the percentage increase in cases in September-December 2020 relative to the same period in 2017–2019 for the eight most common (in 2019) psychological symptoms/diagnoses. All eight increase substantially, especially hyperkinetic disorder (ADHD) and PTSD, about 36% [95% CI 0.30–0.43] and about 33% [95% CI 0.26–0.40].

**Fig. 2:**
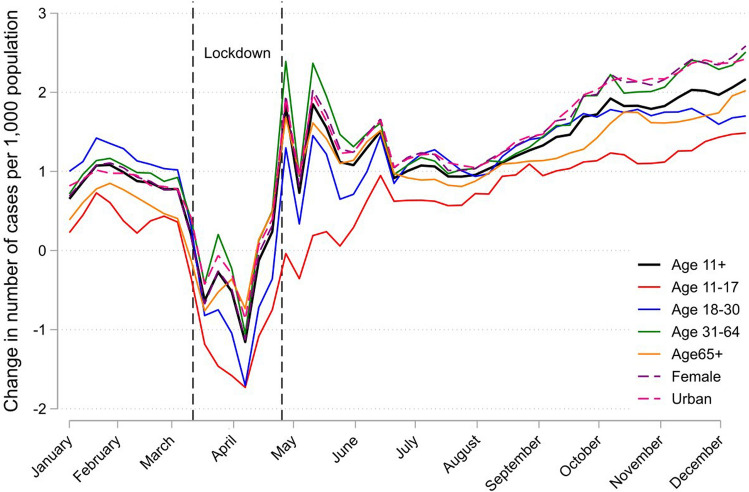
The increase in weekly number of P-cases in 2020 versus 2017–2019 average for subpopulations, per 1000 capita subpopulation. *Note* A “P-case” is a GP consultation that related to a psychological symptom, complaint or diagnosis based on the ICPC-2 classification system (P00–P99). The Figure uses 3-week moving averages for the outcome variables. The leftmost vertical dashed line indicates March 12th, the start of both the first serious outbreak of coronavirus in Norway and the start of national infection control measures, while the rightmost vertical dashed line indicates the end of the strictest measures (e.g. closure of schools and psychologists) on April 27th. Other measures such as social distancing, remote teaching at universities, and remote work were in place throughout most of the period after March 12th. Other measures such as social distancing, remote teaching at universities, and remote work were in place throughout most of the period after March 12th

Figure 2 shows weekly increase of P-cases in 2020 compared to the 2017–2019 average (the shaded area in the top panel of Figure), at a per capita level. The bold line depicts a population-wide weekly increase of about 1 per 1000 capita in June–August, which doubled to about 2 end-of-year. Females, age 31–64, and urban areas experienced the larger per capita increases.^4^

## Discussion

The number of psychological cases in Norway was high relative to prior years in late spring and early summer 2020, consistent with survey evidence from other countries [1–4], but then fell back towards pre-2020 levels during July and August, as depicted in Fig. 1. Our main finding is the acceleration of cases starting September 2020 and still present end-of-year, also depicted in Fig. 1 and Table 1. At a per-capita level, the increase in weekly cases relative to prior years was about 1 per 1000 capita in July–August and doubled to 2 per 1000 capita in December, as depicted in Fig. 2. The acceleration of psychological cases during fall 2020 suggests that the accumulated effects of stress in the fall of 2020 outweighed the development of better coping strategies in the population.

As Norway had low incidence of COVID-19 cases and deaths during fall 2020 compared to many other countries it seems plausible that the acceleration in cases during fall was due to accumulated effects of lockdowns and movement restrictions (rather than stress due to fear of infection).^5^ Our findings should be of interest to policy makers in many countries, who contemplate the difficult trade-offs of continued lockdown policies. Our findings also have broader interest, outside the COVID-19 policy debates, in providing detailed population-level documentation of the mental health effects of prolonged shutdowns and limits to social interaction.

The main cities in Norway have been hubs for COVID-19 cases and lockdowns, as many metropolitan areas globally, and experienced larger increases during September-December than more rural areas, both at a per-capita and percentage level. The increases were also large for females. The adolescents (11–17 age) experienced a large percentage increase relative to other groups (but a lower per-capita increase).

As can be seen in Fig. 1, the number of psychological cases in Norway were unusually high in January 2020. We are not aware of institutional or regulatory changes in 2020 that could explain the spike in January 2020. One explanation is that the unusually foul weather in January 2020 led to a “lockdown” created by nature.^6^ This interpretation is supported by the number of P-cases just before the outbreak in March 2020 being very similar to previous years. In Table 1, we controlled for the possibility of a “2020 effect” unrelated to COVID-19 by comparing the increase in average weekly cases during weeks 40–51 in 2020 to the corresponding increase during weeks 1–10 of 2020 (i.e., prior to the outbreak). The estimate from this approach (Panel A of Table 1) implies that the extra increase in P-cases during weeks 40–51 in 2020 was 9%, i.e., still substantial.

As noted earlier, Norway is characterized by a “long winter” effect, in that the number of P-cases are typically increasing during the fall months [9], possibly due to lack of sun exposure [10]. This can also be seen from Fig. 1 (black line). To investigate whether the long winter effect possibly interacts with the COVID-19 effects, in Panel B of Table 1 we analyzed the increases in P-cases for the three northern-most counties (Nordland, Troms, and Finnmark). The percentage increase, about 9% (95% CI 0.04–0.15), is lower than the increase for the overall population (the first row), which suggest that the long winter effect is not driving our results.

## Supplementary Information

Below is the link to the electronic supplementary material.

Supplementary file 1 (DOCX 159 KB)

## Footnotes

1 https://ehelse.no/kodeverk/icpc-2e--english-version contains more information on ICPC-2, including a mapping to ICD-10.

2 We confine attention to encounter codes 2a and 2e. The other GP encounters in KUHR include tests without patient visits, extra time needed for a consultation (this extra time will be added as a separate row in the data), writing of prescriptions and doctor’s certificates without consultation etc. Other encounter codes than 2a and 2e are usually not included in official statistics by Statistics Norway.

3 Norway has a seasonal pattern in P-cases, in that the number of cases typically increases during the fall months [9], possibly due to lack of sun exposure [10].

4 While the age groups 11–17 and 65 + had the largest percentage increases in P-cases (as shown in Table 1), Fig. 2 shows relatively lower increases in P-cases per capita for these groups, as they had low initial per capita levels.

5 The lockdown policies in Norway can be briefly described as follows. Norway implemented strict national infection control measures from March 12^th^ onwards. Pre-schools, schools, universities, psychologists, physiotherapists, gyms, pubs, etc. were required to close, and non-residents were barred entry to the country. Social distancing rules were also implemented. About April 20^th^, the main infection control measures were relaxed, and pre-schools, schools, physiotherapists and psychologists were allowed to open. Gradually, other measures were relaxed too. However, rules and recommendations restricting social contact and encouraging remote work were in place throughout the period after March 12^th^. From October 26^th^, measures on social distancing, remote work, and the hospitality industry were again tightened.

6 January 2020 was the wettest January ever in Norway. See e.g., https://kommunikasjon.ntb.no/pressemelding/januar-2020-ble-den-vateste-noen-gang?publisherId=17846853&releaseId=17878908
