# Supplementary material for: COVID-19 and mental health: a longitudinal population study from Norway
Source: Eur J Epidemiol. 2022 Jan 27;37(2):167–72. doi: 10.1007/s10654-021-00836-3 (PMC8791672; doi:10.1007/s10654-021-00836-3)
Supplement: Supplementary file 1 — (DOCX 159 KB) [file 10654_2021_836_MOESM1_ESM.docx]

**Supplementary appendix**

**Table S1** shows the fraction of the Norwegian population with at least one GP consultation in 2020, and in prior years, broken down on subpopulations.^[[1]](#footnote-1)^ As shown in **Figure S1**, The Norwegian GP system did not experience a large drop in encounters around the outbreak of the COVID-19 pandemic, relative to prior years, very different from other countries (see Mansfield et al., 2021, for the UK, and Holland et al., 2021, for the US). The reason was a fast transition to electronic encounters. **Figure S2** plots the fraction of consultations that were electronic through 2020.^[[2]](#footnote-2)^

**Table S1. GP Coverage rates in Norway 2017-2020.**

|  |  |  |  |  |  |  | Fraction of Norwegian population with at least one GP consultation in given year | | | |
| --- | --- | --- | --- | --- | --- | --- | --- | --- | --- | --- |
|  | | | | | | | 2017 | 2018 | 2019 | 2020 |
| Age 11+ | | | | | | | 0.76 | 0.75 | 0.75 | 0.75 |
| Age 11-17 | | | | | | | 0.68 | 0.67 | 0.67 | 0.65 |
| Age 18-30 | | | | | | | 0.70 | 0.70 | 0.69 | 0.68 |
| Age 31-64 | | | | | | | 0.75 | 0.75 | 0.75 | 0.74 |
| Age 65+ | | | | | | | 0.87 | 0.86 | 0.86 | 0.86 |
| Male | | | | | | | 0.69 | 0.69 | 0.69 | 0.69 |
| Female | | | | | | | 0.82 | 0.82 | 0.81 | 0.80 |
| Urban | | | | | | | 0.82 | 0.83 | 0.83 | 0.79 |
| Rural | | | | | | | 0.73 | 0.73 | 0.72 | 0.73 |
|  | | | | | | |  |  |  |  |
| Population size (million) | | | | | | | 4.57 | 4.61 | 4.65 | 4.70 |


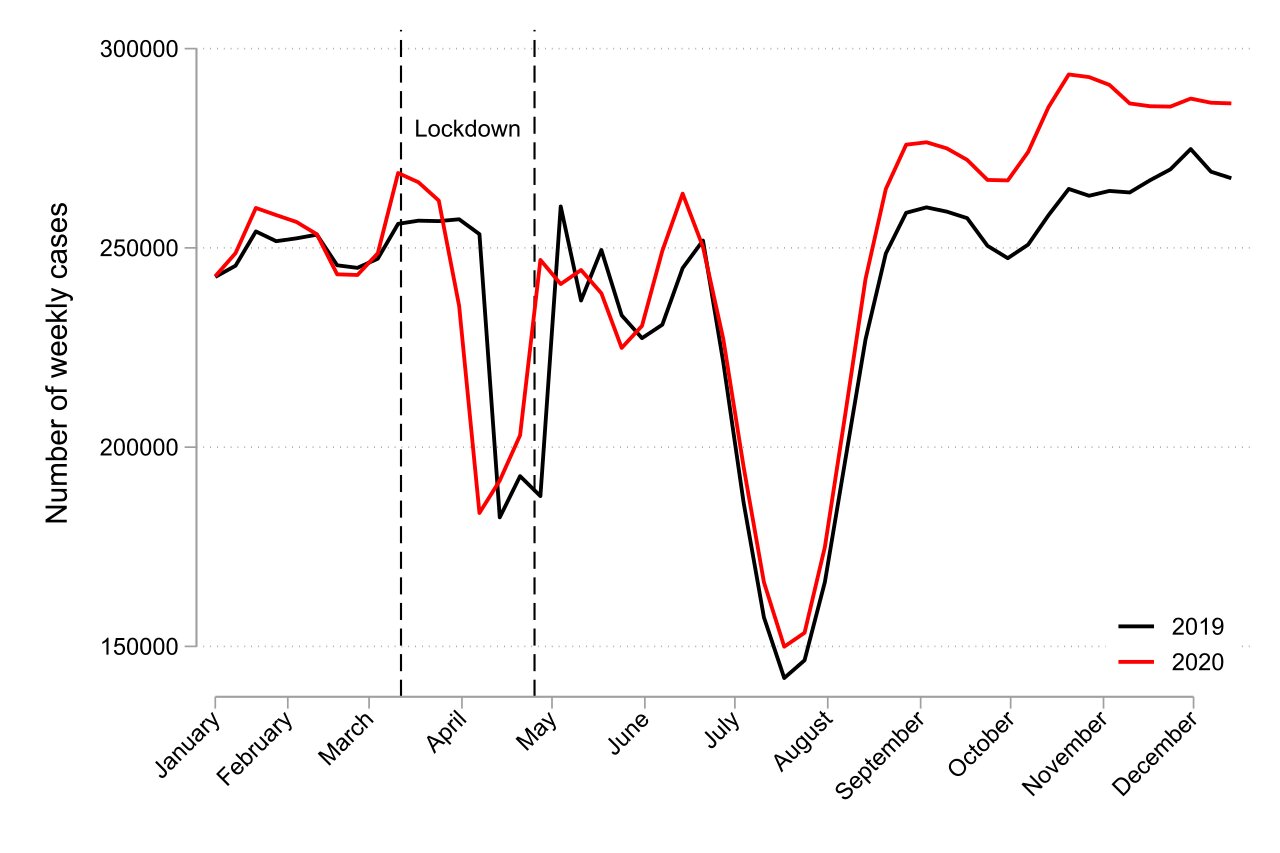


**Fig S1. Number of weekly GP consultations in 2020 (red) versus 2019 (black)**

*
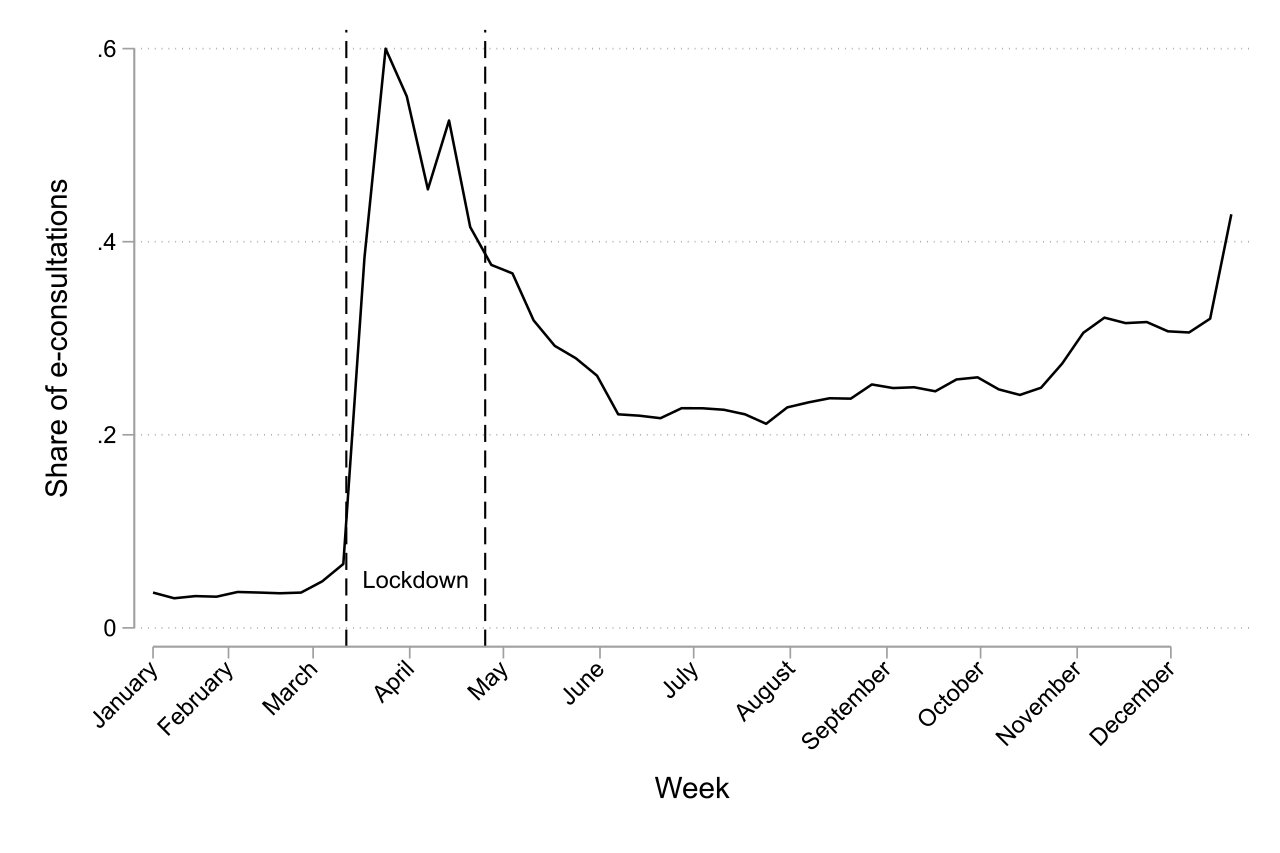
*

**Fig S2. E-consultations as fraction of all consultations, by week of 2020**

1. The Norwegian population totals are from Statistics Norway (www.ssb.no). [↑](#footnote-ref-1)
2. The homepage of The Norwegian Medical Association contains additional information on the GP refund arrangement, and the transition to electronic consultations during 2020 in Norway. <https://normaltariffen.legeforeningen.no/book/Fastlegetariffen-2020/m-02>. [↑](#footnote-ref-2)
